# The preferences of users of electronic medical records in hospitals: quantifying the relative importance of barriers and facilitators of an innovation

**DOI:** 10.1186/1748-5908-9-69

**Published:** 2014-06-05

**Authors:** Marjolijn HL Struik, Ferry Koster, A Jantine Schuit, Rutger Nugteren, Jorien Veldwijk, Mattijs S Lambooij

**Affiliations:** 1 Health Sciences, specialization Policy and Organization of Health Care, VU University Amsterdam, Amsterdam, The Netherlands; 2 Department of Sociology, Erasmus University Rotterdam, Rotterdam, The Netherlands; 3 Department Quality of Care and Health Economics, National Institute for Public Health and the Environment (RIVM), Center for Nutrition, Prevention and Health Services, Bilthoven, The Netherlands; 4 University Medical Center Utrecht, Julius Center for Health Sciences and Primary Care, Utrecht, The Netherlands; 5 National Institute for Public Health and the Environment (RIVM), Bilthoven, The Netherlands

**Keywords:** Electronic Medical Record, Implementation, Discrete choice experiment, Adopter preferences, Barriers and facilitators, Clinicians

## Abstract

**Background:**

Currently electronic medical records (EMRs) are implemented in hospitals, because of expected benefits for quality and safety of care. However the implementation processes are not unproblematic and are slower than needed. Many of the barriers and facilitators of the adoption of EMRs are identified, but the relative importance of these factors is still undetermined. This paper quantifies the relative importance of known barriers and facilitators of EMR, experienced by the users (*i.e.*, nurses and physicians in hospitals).

**Methods:**

A discrete choice experiment (DCE) was conducted among physicians and nurses. Participants answered ten choice sets containing two scenarios. Each scenario included attributes that were based on previously identified barriers in the literature: data entry hardware, technical support, attitude head of department, performance feedback, flexibility of interface, and decision support. Mixed Multinomial Logit analysis was used to determine the relative importance of the attributes.

**Results:**

Data on 148 nurses and 150 physicians showed that high flexibility of the interface was the factor with highest relative importance in their preference to use an EMR. For nurses this attribute was followed by support from the head of department, presence of performance feedback from the EMR and presence of decisions support. While for physicians this ordering was different: presence of decision support was relatively more important than performance feedback and support from the head of department.

**Conclusion:**

Considering the prominent wish of all the intended users for a flexible interface, currently used EMRs only partially comply with the needs of the users, indicating the need for closer incorporation of user needs during development stages of EMRs. The differences in priorities amongst nurses and physicians show that different users have different needs during the implementation of innovations. Hospital management may use this information to design implementation trajectories to fit the needs of various user groups.

## Background

Diffusion of innovations results from the interaction between technology producers, users, and external groups and the system adopting an innovation [1,2]. Although user acceptance of an innovation varies between individuals, studies found several barriers and facilitators that generally apply to the implementation of innovations [2-6]. Knowledge about these barriers and facilitator is integrated in several overlapping theoretical frameworks [7]. Barriers and facilitators of implementation in organizations can be found on the user level (*e.g.*, preferences and skills), the organizational level (practical support and culture), or on the level of the innovation (ease of use, added value to users) [2,3,6].

Clearly, a well-designed implementation plan uses facilitators and overcomes barriers in the different phases of the implementation process. The process begins with the proposition to initiate change, moves to evaluation and re-adjustment of the implementation plan, then needs to involve aspects of the innovation/change, of the stakeholders and of the (organizational) context of the implementation process [7].

In this paper we focus on one phase in this implementation process of a specific innovation: the user acceptance of electronic medical records (EMRs) in Dutch hospitals. Since EMRs are currently implemented or improved in a majority of Dutch hospitals, they provide a unique opportunity to evaluate the preferences of users. We will use their responses to investigate and order their preferences. Building on knowledge from prior implementation studies, we aim to gain insight in the relative importance of the added value of the innovation, the ease of use of the innovation and the organizational support in the support for the innovation by its users [2,6,8].

Quantifying the relative importance of those factors, in the preference of users, enables identification of the most prominent problems that users face in the current stage of the implementation process. This knowledge may help developers, implementers, and policy makers to design and adapt implementation trajectories, giving attention to the most relevant issues as perceived by the users.

We test the relative importance of these factors using a discrete choice experiment (DCE). A DCE is a quantitative method of eliciting preferences concerning a good or service, which is an up-coming method in public health to determine what factors influence people’s willingness to participate in and pay for medical or preventive interventions [9-11].

Two articles [12,13] used conjoint or DCE techniques to address implementation related issues. The first study used conjoint analyses to elicit preferences of stakeholders concerning innovations in general. That study concluded that a conjoint analysis is useful in studying the preferences of health care professionals [12]. The second study used a DCE^a^ to prioritize barriers among physicians for the use of a breast cancer guideline. That study concluded that it was far too time consuming to let physicians prioritize all options [13]. Given the contradicting conclusions of these studies, the current study has a complementary aim to explore the use of DCEs in implementation studies amongst health care professionals.

To realize the potential benefits (eg in communication, patient management, research and patient safety [14]; structuring the health care process, health care costs reduction [15], and improvement of quality [16], efficiency and safety [17]) clinicians need to incorporate EMR usage in their daily practice. The more accurate, concise and timely the patient data are entered into the system, the more beneficial the system will be for all its users. Since physicians and nurses showed positive attitudes towards the EMR after using it for a while [17], nothing seems to slow down the implementation of this innovation. However, despite that some studies show positive attitudes and expectations, the speed of the adoption remains slow [16,18] and the main cause for this slow speed is not always clear.

**Figure 1:**
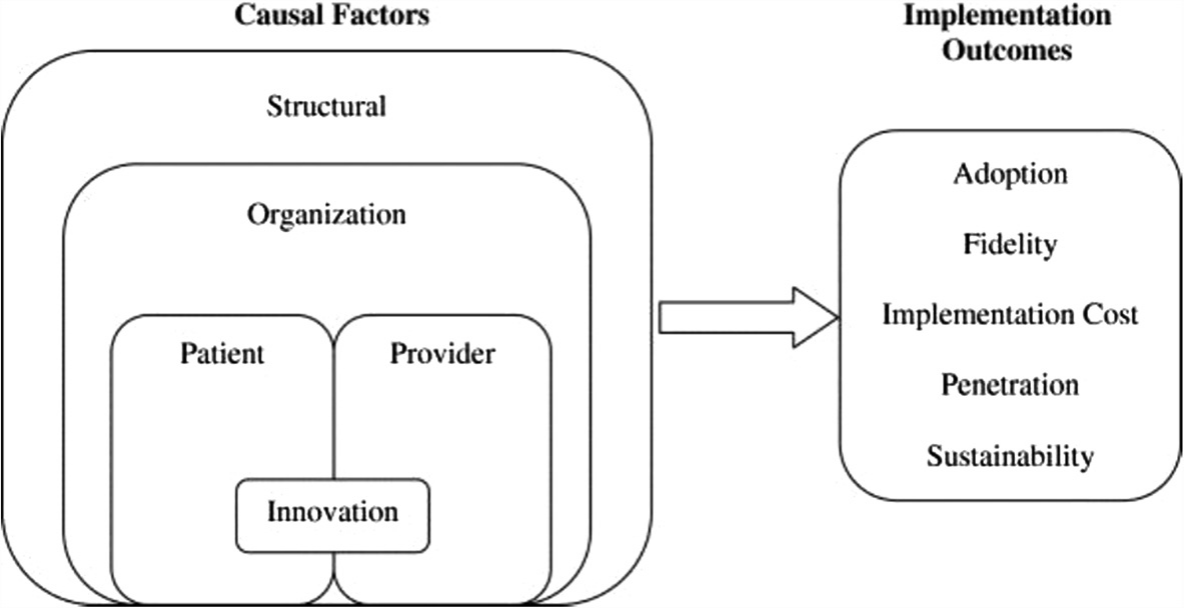
**A multi-level framework predicting implementation outcomes **[3]**.**

A systematic ordering of factors investigated in implementation studies reveals that most studies investigate factors on the user level, the organizational level or on the level of the innovation (Figure 1) [3]. The structural level and patient-level factors were studied to a lesser extent. Instead of taking all these factors into account, the present study focuses on the relative importance of two of the most studied levels, namely the innovation and the organizational. This choice is not intended to downplay the importance of other factors, since social, cultural, and political factors are expected to play a role in the EMR implementation process, but here we aim at investigating the two core factors in more detail than earlier studies. While they have been studies before, their relative importance remained unknown. The second reason to focus on these aspects is that in the case of the EMR, the social, and political factors are to a large extent similar to all Dutch employees, making it impossible to include in this study. Third, patient level factors are not taken into account because we are mainly interested in the preferences of the users (*i.e.*, clinicians) who apply an EMR in the care for all patients, and patients do not use EMRs.

The research question is: What is the relative importance in the preference of users of EMRs in hospitals of factors that are related to the innovation itself (perceived benefits and ease of use) and organizational context (support from other organizational departments) for users (physicians and nurses) of EMRs in hospitals?

## Methods

### Discrete choice experiment

We used a Discrete Choice Experiment (DCE) to quantify the relative importance of the selected factors for the willingness to use the EMR of two users groups, physicians and nurses in Dutch hospitals. DCE is a method to elicit individuals’ preferences concerning a service or good [9,10,19,20]. In a DCE respondents are asked to react on a presented scenario. In the current study, we selected respondents who have experience with working with an EMR in their current position. Therefore, little introduction to the scenario was necessary. The question prior to the presentation of the scenarios was: ‘Please indicate in which of the following situations you prefer to use the electronic medical record’.

Subsequently, a number of choice sets of two scenarios are presented to the respondents. The scenarios are described by different characteristics of the service or good (*i.e.*, attributes). Every attribute contains several levels, representing different values. In this study the attributes are the factors that we expect to affect the willingness to use the EMR, and the levels are varied.

Respondents choose the scenarios based on the differences in the levels of the attributes. The DCE is based on Random Utility Theory, which assumes that respondents choose the scenario with the highest personal utility [9,20], whereby utility U exists of a predictable component V and an unpredictable component ϵ (U = V + ϵ) [21]. The answers of the respondents to multiple choice sets enables researchers to estimate the relative importance of different attributes of the scenarios [10,11,20].

### DCE design

NGene 1.1 software (Choice Metrics, 2011) was used to construct the choice sets of this DCE. A D-efficient design was used for this procedure [22]. This results in a minimal number of choice sets that have to be completed by a minimal number or respondents in order to be able to detect significant differences.

In total, 10 unique choice sets were constructed for this DCE (see Additional file 1 for a choice set example). Each choice set consisted of two scenarios, subsequently asking the respondents: ‘Please indicate in which situation you prefer to use the electronic medical record’ After choosing one of the scenarios respondents were asked to indicate whether they would indeed use this type of EMR or would rather not use it (opt-out). We chose this method to measure the opt-out for a number of reasons. First, the use of an EMR in a hospital is not a real choice for the clinicians. So including an opt-out would not be congruent with the real life situation. Second, we wanted to know how willing the respondents where to use an EMR *per se*. By adding the additional question, we gathered information on the willingness of the respondents to use their EMR. Third, this way of measuring the opt-out has the advantage that the information of the trade offs are measured (this information is lost in a conventional opt-out) and we have information whether the respondents would rather opt-out. Before the choice sets were presented to the respondents, all attributes and levels were explained and at any choice set respondents had the possibility to see the explanations once more (Additional file 2). Additionally, it was explained how the choice sets should be answered, including an example.

### Attributes and levels

In the design of a DCE, it is crucial to find a balance between a realistic scenario that includes the relevant factors and the complexity of the design, to avoid cognitive overload of the respondents. We therefore limited the number of attributes to six per scenario. Three of the attributes are on the level of the innovation and three are on the level of the organization.

The attributes were based on existing literature and pretested in short interviews with users of EMRs [2,5,6,23]. Subsequently the DCE was piloted among fifteen nurses and doctors. It was tested whether they understood the questions and if they could relate the scenarios to their own situation. This did not lead to changes in the design.

### Attributes on the innovation level

On the level of the innovation, we included three attributes, Data entry hardware, flexibility of the interface and the presence of decision support.

The attribute data entry hardware contains two attribute levels:

Computer/workstation: You are able to enter and request patient data on a number of fixed locations (*e.g.*, at outpatient clinic, operating theater and your office).

Tablet: You are able to walk in the hospital and update and request patient data at any desired location.

This attribute was selected because, ease of use and added value to the user are expected to positively affect the willingness to use an innovation [8,23-26]. If EMR systems were user-friendly, and compatible with the working environment, users perceived them as easy to use and valuable to facilitate work processes and were more willing to implement the system than when the system was unclear and less compatible to the work environment [4,27].

The levels were chosen to vary the ease of use for the users. Recent studies have shown positive attitudes of physicians towards the use of tablets, because of its simplicity, similarity to paper and intuitive nature [28,29]. Tablets have shown to enhance interactions between physicians and patients and improve their workflow because information is easier accessed and physicians have increased mobility [28].

Flexibility of the interface of the EMR is related the ease of use of the innovation. We varied the following levels:

Low: The system provides a comprehensive overview of all medical information. You have to scroll to see relevant information.

High: You can configure what information is displayed at first (*e.g.*, patient data, lab, medication, imaging, diagnosis etc).

This attribute can also be linked to and efficacy (compatibility) of the innovation [2]. A study indicates that nurses were frustrated when they did not gain useful data from an EMR system, while they knew that EMR systems could assist them with patient care by providing in rightful information [30]. Systematic reviews show evidence for that when the EMR is not adapted to needs or abilities of its users, the EMR is perceived as difficult to use [27], causing a user to not adopt the EMR [18].

The third attribute on the level of the innovation is the presence of decision support in the EMR. The two attribute levels are:

Absent: the system has no alarm for risk situations.

Present: with sound and image you will be warned for risky situations (*e.g.*, extreme medication dosage or potential medication interactions).

Decision support is designed to aid clinicians to avoid mistakes and may therefore improve job performance [23] and as such have added value in terms of productivity and efficiency [27] for the users, increasing the chance of adoption [4]. The decision support system can alert or remind users in case of adverse drug events, for example overmedication or antagonizing of different drugs. The reminders and alerts can warn a user when changes are made in a patient’s EMR, in turn this may increase patient safety [17]. There is also some evidence that decision support may improve the care process in chronic disease management and patient health [31]. Also, decision support can modify behavior of physicians in ordering tests [32]. These features add value to the current situation and might consequently lead to gains in job performance.

### Attributes on the organizational level

On the level of the organization we also included three attributes in the DCE. They are related to practical support, managerial support coherence and added value.

The attribute technical support has the following levels:

IT helpdesk: the regular hospital-wide helpdesk can be called for all kinds of IT problems, including issues in EMR.

Training: The hospital ensures that a certified trainer shows you in a one day training what the system can do. When subsequently using the system, you can use the regular helpdesk.

A number of studies found that organizational and technical factors influence the implementation of EMRs [3,4,16,18,27,33]. Technical support covers the belief of the existence of an organizational and technical infrastructure that support the use of the system [23]. In Rogers’ theory of diffusion, it is linked to the triability and observability of an innovation [2].

Lack of training and technical support hinders the EMR implementation [18]. Training in using the EMR has shown to affect nurses’ attitude towards the EMR positively [34]. We therefore expect that when implementation is flanked by training, the users are more willing to use the innovation.

Managerial support in the organization is also expected to affect the implementation and was included with the following levels were presented in the DCE:

Biding: The head of department emphasizes that the use of the EMR should interfere the regular work as little as possible.

Stimulating: The head of department emphasizes the importance of good use of EMR for the quality of the work of the department.

Managerial attitudes have shown to affect the success of the implementation in case of EMRs [18], but also in other innovations [35,36] in several ways, for instance in information diffusion and by developing strategies regarding to innovations [23,37]. Research of Grol *et al.* show that implementations improve if managers have a stimulating attitude towards an innovation [5,6]. In case of EMR, it may be expected that a stimulating attitude of the head of department leads to more acceptance.

The final attribute on organizational level relates to organizational use of the information that becomes available after implementation of the EMR

This attribute consisted of the following levels:

No overview: You do not receive any information from the EMR concerning your department.

Monthly overview: You receive a monthly e-mail with an overview of your department with number of patients, mean age of patients, number of diagnoses, number of complications, and satisfaction ratings of patients.

When the organization uses the innovation to give the employees systematic feedback on performance, that they did not have before the implementation, this is showed to be valued by clinicians [38-40]. We therefore expect that when the implementation of the EMR is accompanied with systematic feedback on performance of the department, this will positively affect the willingness to use the EMR.

### Participants

The respondents consisted of physicians (medical specialists and medical residents) and nurses (head nurses, specialist nurses, regular nurses and other) of different hospitals in The Netherlands. Participants were derived from internet survey panels via external research agencies. 311 nurses and 550 physicians were contacted to participate. The respondents could fill out the questionnaire via the internet. Respondents who completed the questionnaire received a monetary reward.

According to The Dutch National Ethics Board (Central Committee on Research involving Human Subjects) formal testing by a medical ethical committee was not necessary because the responding clinicians were asked to fill out a 1) single, anonymous survey that 2) did not include radical, incriminating or intimate questions and 3) filling out the survey did not require much time. This is in accordance with the guidelines laid down in the Declaration of Helsinki.

### Statistical analyses

In nLogit 4.0 (Econometric Software Inc. 2007), Panel Mixed Multinomial Logit (Panel-MIXL) analyses were performed for the data of physicians and nurses separately. This statistical method enables to adjust for the dependency of observations among individuals (*i.e.*, every respondent answered multiple choice sets). Levels of attributes were effects coded because of their non-linearity. In contrast to dummy-coding, with effects-coding it is made possible to compare the different attributes with each other with respect to their relative importance [41]. The attribute estimates for the reference category (coded as -1) can be calculated by multiplying -1 with the estimate of the attribute (see Table 1 for coding).

**Table 1:** Attributes, coding in analyses, and link with hypotheses

**Attribute**	**Attribute level and coding**	**Level of implementation and aspect**
Data entry hardware; hardware to access EMR	-1 Computer/workstation	Innovation level, ease of use
	1 Tablet	
Flexibility of interface; user can tailor to wishes, versus static	-1 Static: need to scroll	Innovation level, ease of use
	1 Flexible: user is able to tailor set desired information	
Decision support in EMR or not	-1 No decision support present	Innovation level, added value
	1 Decision support present	
Practical support, regular IT helpdesk or supported by training	-1 Regular IT support	Organization level, practical support
	1 IT support, combined with training	
Attitude of your manager; stimulating or biding	-1 Biding	Organization level, managerial support
	1 Stimulating	
Performance feedback; monthly overview of performance department	-1 No performance feedback	Organization level, added value
	1 monthy overview performance department	

Alternative specific constants (ASCs) were included for alternative A and B. Since these constants significantly differed from each other in the physicians’ data, no generic constant could be included. To perform uniform analyses, in analysis of nurses ASCs were used as well.

The utility regression equations were described as following:

$$\begin{array}{l} U=\left(\mathrm{ASCC}\right)+\beta 1*\left(\mathrm{data}\;\mathrm{entry}\;\mathrm{by}\;\mathrm{tablet}\right)\\ \;+\beta 2*\left(\mathrm{technical}\;\mathrm{support}\;\mathrm{incl}\;\mathrm{training}\right)\\ \;+\beta 3*\left(\mathrm{attitude}\;\mathrm{head}\;\mathrm{of}\;\mathrm{department}\;\mathrm{stimulating}\right)\\ \;+\beta 4*\left(\mathrm{performance}\;\mathrm{feedback}\;\mathrm{in}\;\mathrm{EMR}\right)\\ \;+\beta 5*\left(\mathrm{flexibility}\;\mathrm{high}\right)\\ \;+\beta 6*\left(\mathrm{decision}\;\mathrm{support}\;\mathrm{in}\;\mathrm{EMR}\right)\\ U\left(\mathrm{opt}-\mathrm{out}\right)=0 \end{array}$$

The need for random parameters were tested in this model based on model fit. We tested whether including different attributes as random parameter (assuming normal distribution) resulted in a significant better model fit based on AIC and log-likelihood. The final model contained no random parameters, except for the intercept. The answers were coded as opt-out when respondents had answered that that would prefer not to use the EMR in either scenario of the choice set.

Outcomes of analyses were different attribute estimates (beta values), corresponding standard errors and p-values calculated with the use of Z-tests, used significance level was 0.05. Negative estimates imply that respondents prefer the reference level of the attribute. With these attribute estimates a ranking of attributes was made, from high (highest relative importance) to low (lowest relative importance).

## Results

### Sample

In total, 148 of 311 nurses (47.6%) and 150 of 550 physicians (27.3%) completed the questionnaire, Table 2 presents the descriptive statistics of the sample. Gender distribution and mean ages of the sample were compared with population data obtained by Statistics Netherlands [41]. Mean age of male nurses (16% of all nurses in this study) was 42.5 (s.d. = 11.6), female nurses (84%) had a mean age of 40.1 (s.d. = 11.7). Gender distribution was comparable to nurses’ population in the Netherlands (15% male versus 85% female), only mean ages were somewhat lower in our study population. In Dutch population of nurses in 2010, mean ages were 47.7 for male nurses and 43.4 for female nurses [41,42].

**Table 2:** Descriptive statistics of respondents

	**Nurses (n = 148)**		**Physicians (n = 150)**	
	**Male (n = 23) (16%)**	**Female (n = 125) (84%)**	**Male (n = 109) (73%)**	**Female (n = 41) (27%)**
**Mean age, year (SD)**	42.52 (11.58)	40.14 (11.71)	48.26 (9.64)	43.90 (7.72)
**Number of years working in current job**	9.71 (9.61)		12.73 (9.05)	
**Reported type of hospital**				
University	5	26	19	16
General hospital with teaching facilities	5	32	40	8
General without teaching facilities	9	55	45	14
Peripheral	0	1	0	1
Independent treatment center	0	2	3	2
Other	4	9	2	0
**Profession**				
Medical specialist			103	38
Medical Resident			6	3
Head Nurse	0	10		
Nurse	15	88		
Nurse specialist	6	24		
Other	2	3		

The category ‘other’ exists of a consulting assistant and employees of surgery department, anesthesia, and general support function.

For the 150 physicians our study, mean age for men was 48.3 (SD 9.6) in our study, which was corresponding with the average age of Dutch population of male physicians (48.4). Mean age for female physicians was a little higher in our study, namely 44.4 (SD 8.2) compared to 40.6 in Dutch population of female physicians. Gender distribution of the physicians in the study differed from the national population. In this study there were relatively more male physicians that did respond to the survey compared to the overall physicians’ population of 2010 (54% male versus 46% female physicians) [41]. When performing stratified analyses (not presented), the relative importance of attributes was not different between the genders.

Respondents were working in different types of hospitals, *i.e.*, university hospitals, general hospitals, and independent treatment centers. Nurses that participated in this study were mainly regular nurses or nurse specialists. The group of physicians consisted of medical specialists and residents.

Representativeness of study population was checked on gender distribution, age, profession and reported type of hospital. Based on the information presented above, we concluded that our population was a good reflection of overall population of nurses. We were able to include relatively fewer female physicians compared to the national physicians’ population. However, tests reveal no systematic different answer patterns between the genders.

Outcomes of Panel-MIXL analyses were estimates for the different attributes. With these attribute estimates a ranking of attributes was made, from high (highest relative importance) to low (lowest relative importance).

### Analyses

Results of the Panel-mixed model are presented in Table 3 (physicians) and Table 4 (nurses). In both models the intercepts were set random, controlling for the panel structure of the data and all predictor variables were fixed. For the statistically significant parameters, the size of the parameter indicates the relative importance in the preference of the respondents. The nurses chose opt-out in 23.2% of their answers, the physicians chose opt-out in 21.4% of their answers. The intercept in the model of the nurses is positive and significant (Table 4), and in the model of the physicians (Table 3) the intercept is positive but not significant. The sd’s of the intercept are not significant, but we see that the size of the sd for the physicians (Table 3) is larger than in the model of the nurses. This means that the nurses had a smaller variance in their answers than did the physicians.

**Table 3:** Results of panel-MIXL analyses, for the physicians; dependent variable is chosen scenario

**Attribute**	**Levels (-1 value vs 1 value)**	**Parameter**	**s.e.**
Flexible interface	Need to scroll vs Personal tailor option	0.43	0.04**
Decision support present	No vs Yes	0.25	0.04**
Feedback performance	No feed back versus monthly feed back	0.20	0.04**
Attitude head of department	Biding vs Stimulating	0.18	0.04**
Practical support, including training	Regular helpdesk vs Helpdesk and training	-0.10	0.01**
Data entry hardware	Workstation vs Tablet	-0.10	0.04*
Intercept		0.67	0.39
Sd intercept		0.87	0.93

**p < 0.01; *p < 0.05.

**Table 4:** Results of panel-MIXL analyses, for the nurses; dependent variable is chosen scenario

**Attribute**	**Levels (-1 value vs 1 value)**	**Parameter**	**s.e.**
Flexible interface	Need to scroll vs Personal tailor option	0.35	0.03**
Attitude head of department	Biding vs Stimulating	0.23	0.04**
Decision support present	No vs Yes	0.22	0.04**
Feedback performance	No feed back versus monthly feed back	0.21	0.04**
Practical support	Regular helpdesk vs Helpdesk and training	-0.05	0.04
Data entry hardware	Workstation vs Tablet	-0.17	0.04**
Intercept		0.38	0.10**
Sd intercept		0.13	1.06

**p < 0.01; *p < 0.05.

A high flexibility of interface of the EMR turns out to have highest relative importance in both analyses. In sample of physicians, decision support was the second largest beta, whereas for the nurses the stimulating attitude by the head of department had the second largest beta. Subsequently, performance feedback in the system was found to be important. Attribute estimates were negative for data entry hardware and technical support, which means that respondents preferred workstation above tablet as data entry hardware and, respondents preferred just to get support of the IT helpdesk above an additional training. Technical support and data entry hardware were perceived by physicians and nurses as relatively least important. In both analyses technical support did not yield significant results.

## Discussion

This study investigated the preference structure towards the use of EMRs in the daily practice of work in a hospital of nurses and physicians by quantifying the relative importance of several aspects in the use of the EMR. Because physicians and nurses work in different situations in hospitals, we expected to find differences in their preference structures. In this study we both found similarities and differences in relative importance of attributes perceived by physicians and nurses. These differences can be understood from the different positions in the hospitals. The jobs of nurses are more integrated in the organization’s bureaucratic structure, whereas physicians work more autonomously, possibly explaining the expected result that the attitude of the head of department was more important to the nurses than to the physicians.

The three most prominent factors that the physicians responded to were related to ease of use and added value of the innovation. The first two (flexible interface and decision support) are innovation-level factors and need to be addressed by the producers of the EMR. The third, feedback on performance, can be organized by the hospital.

Even though the results of the nurses show many similarities with those of the physicians, it also shows that nurses attach more value to the organizational support in their preference to use an innovation. After considering the ease of use, managerial support was considered in their preference to use the EMR. After this, the added value of the innovation was considered.

Implications for the implementation practices is that for physicians the innovation need to be fully developed and have immediate ease of use and added value, while for the nurses, the managerial support can positively affect the implementation process amongst the users.

The shared preference of nurses and physicians of a flexible interface may be interpreted as a signal that user friendliness of the current systems may be improved. The finding in this study is in line with previous research [18,27], and with finding of the Health Care Inspectorate that reported that crucial information is not always immediately retrievable, possibly because of hard to read EMR. The inspectorate advises that the information need of users plays a central role in the development of EMRs [43]. That this advice is still applicable is also echoed in an essay question that included in the questionnaire that contained the current DCE. A majority of respondents that had used to opportunity to answer this question, referred to the cumbersome nature of their EMR that causes inefficiency and inconvenience.

Decision support was also found to be important by both groups. Decision support warns users of EMRs for risky situations. Currently, decision support is not often an integral part of EMR. If the expectations by Gartner on future EMRs become true, somewhere in the future, this feature will become standard. Based on the outcomes in this study, we expect that generation of EMRs to be embraced more heartily by the users than the current generation.

Both physicians and nurses preferred monthly performance feedback above no feedback, which was ranked third in both groups. With this monthly overview of patient characteristics, number of diagnoses, complications and patient satisfaction scores, it is possible to motivate physicians and nurses change their usual practice when they receive reports of inferior or inconsistent care [38]. Consequently, this will result in an improvement of quality of care, delivered by that physician or nurse, which also affects the overall quality of health care. Physicians do appreciate this attribute; this might imply that they are willing to improve the quality of delivered care. This is a potentially useful outcome for policy makers who are involved in improving health care, but also for implementers of the EMR. A performance feedback system can make clinicians aware of their delivered quality of care and thereby possibly contribute to the improvement of quality of care.

Against expectations, nurses and physicians preferred the use of computers or workstations above tablets. This contrasts with evidence in literature suggesting that nurses and physicians prefer the use of tablets instead of workstations, because of various reasons. On the one hand, tablet use may enhance the patient-caregiver interaction when doctors are not sitting behind a desk watching the computer screen, but use a hand-held device when consulting patients [28]. But on the other hand, another study reported that smartphone and tablet use in hospital settings may have several negative effects, such as distraction by text messaging, unprofessional behavior, infection or hygiene risks, and interference with other medical equipment [44]. It is unclear why respondents in our study did not prefer the use of tablets. A potential cause can be the fact that clinicians are happy in the current situation where they are working with computer or workstations. Many EMR contain multiple tabs, and this may be easier to operate from a desktop computer. Ease of use may also apply to writing additional information in text, because writing text with a tablet is less comfortable than typing on a keyboard. Choosing for the tablet for data entry means a new technique and thus a change of behavior and higher costs of the innovation. When using the ‘old-fashioned’ workstations for data entry in the EMR, users do not need to dramatically change their behavior, which leads to lower costs compared to when using Tablets.

The second unexpected result is finding that the respondents do not prefer to get training for the EMR. Several studies have shown the importance of training and technical support [18,27,33,34]; training in using the system has shown to influence the attitude of nurses positively [34]. Also, training in the system and adequate technical support might be necessary to counteract resistance of using EMRs [18,27,42]. In the scenario, we stated that the training would take a day. This may have generated a response we did not anticipate in advance. It implies that the added value of the training costs a day of valuable time, maybe even spare time, because this was not clearly explicated in the questionnaire. We assume that the wording in our design is responsible for the relatively low priority given to this factor.

Concerning the use of DCE’s in implementation studies, a number of conclusions can be drawn after this study. Previous studies lead to contradictory conclusions on using DCEs in implementation research. The most critical of these studies stated that DCEs were less suited to elicit preferences from physicians based on a response rate of 10% [13]. The response rate in this study was 27% for physicians and 48% for nurses. This means that we also found that physicians are harder to recruit, however, in the description of our sample we presented mean age and percentages of genders. In the nurses sample, we found no substantial deviations between the national statistics and our sample. The sample contained fewer female physicians than in the national population, however we found no evidence that female respondent physicians had structurally different answer patterns. This under representation of female physicians could be because female physicians are less interested in the topic of research. We cannot exclude that on average the female physicians had answered differently if they were would have been included. A second indication of less added value of the used methods would be that respondents stopped seriously answering the questions and answered the questions without reading them. This may have shown up in respondents answering the scenario on the right side (or left side) of the screen. However, we tested for this left to right bias and found no evidence of this. Third we studied the answers of the respondents to the final question in the questionnaire whether they had comments on the questionnaire. We found there that two physicians and five nurses objected to this way of questioning. On the other hand, two nurses praised the questionnaire. Finally, the plausibility and understandability of the majority of the answer patterns lead us to conclude that this DCE has generated valuable insights in the preferences of physicians and nurses and that this method can also be considered as a tool to study the preferences of users for other innovations.

The strong preference for the flexible interface for both user groups may be a signal that the reported preferences are influenced to the moment of answering the DCE. Given the findings of earlier research and of the inspectorate, many of the EMRs currently used in Dutch hospitals are not optimally suited to give health care professionals a quick and easy access to the information that they need. The current study asked the respondents to refer to their own situation. The importance that they attach to the interface may therefore be caused by current frustrations of their own EMR. If this study will be replicated later on, when a new generation of EMRs is implemented, we expect the order of relevance to be different. The fact that we could not correct for the fact that implementation stage is likely to affect the preferences of the users [45], is a limitation of this study.

In this study, internet panels were used to approach respondents. Quality of responses of internet panels were already described by Chang and Krosnick, internet panel members will give more reliable answers on attitude questions when they become more experienced in responding to them [46]. Nonetheless, experienced completing of questionnaires may also lead to ‘panel conditioning.’ This implies that an increasing experience in doing surveys makes members less representative of the population they were derived from. However, no evidence or only very small effects were found when studying this subject. Also, sample composition bias caused by interest in the topic may be present, because those who are interested in the topic might be more likely to participate in the survey [47,49]. However, this problem may be unavoidable in every form of research that depends on respondent participation.

## Conclusions

To our knowledge, this is the first study where relative importance of barriers and facilitators of EMRs in hospitals are quantified among clinicians. DCEs are used in other fields of research, but it is relatively unknown in health care based implementation literature. However, prioritizing the barriers and facilitators for other types of interventions may give valuable information for people designing implementation strategies. Although it is a snapshot in time, policymakers, implementers and developers of the EMR may use the information to fine tune implementation processes and anticipate the preferences of the users and future implementation processes. This study shows that for the nurses, managerial support will be more effective in promoting acceptance of the EMR than for physicians. It also shows that the physicians are mainly concerned with the ease of use and added value of the innovation. This means that during the implementation the EMR has to function flawlessly directly and should be easy to work with, in order to gain support from the physicians. The hospital will positively affect the support of the physicians if they use the information of the EMR for performance feedback on departmental level. For the physicians, the key to innovation acceptance lies within added value and ease of use, while for the nurses the organizational context can help to affect their acceptance for the innovation.

## Endnotes

^a^Although a DCE and conjoint approach differ methodologically [14,47], they both address the same type of questions and they both enable eliciting the relative importance of included attributes.

## Abbreviations

DCE: Discrete choice experiment; EMR: Electronic medical record; IT: Information technology.

## Competing interests

All authors declare to have to competing interests

## Authors’ contributions

MHLS wrote the concept of the main body of the text and contributed to the analyses. FK contributed in the conceptual work of the text and critically reviewed text and analyses. AJS contributed in the conceptual work of the text and critically reviewed text and analyses. RN contributed to the acquisition of the data and reviewed the text. JV contributed to the design and interpretation of the analyses. MSL developed the research framework and data collection, supervised data acquisition, co-wrote the main body of the text and conducted analyses. All authors read and approved the final manuscript.

## Supplementary Material

Additional file 1 Example choice set. Click here for file

Additional file 2 Instruction sheet for respondents. Click here for file
