# Supplementary material for: The preferences of users of electronic medical records in hospitals: quantifying the relative importance of barriers and facilitators of an innovation
Source: Implement Sci. 2014 Jun 5;9:69. doi: 10.1186/1748-5908-9-69 (PMC4088913; doi:10.1186/1748-5908-9-69)
Supplement: Additional file 1 — Example choice set. [file 1748-5908-9-69-S1.docx]

**Additional file 1. Example choice set**

|  | **Sitation 1** |  | **Sitation 2** |
| --- | --- | --- | --- |
| Data entry | Tablet |  | Computer/workstation |
| Practical support | Training |  | IT helpdesk |
| Attitude manager | Biding |  | Stimulating |
| Feed back | Monthly overview |  | No feed back |
| Flexibility | Static |  | Flexible |
| Decision support | Not present |  | present |
| I prefer to use the system in | Situation 1 |  | Situation 2 |

Which answer applies?

1 also in the chosen situation I prefer not to use the EMR

2 In the chosen situation I will use to EMR with no objection
