# Supplementary material for: The preferences of users of electronic medical records in hospitals: quantifying the relative importance of barriers and facilitators of an innovation
Source: Implement Sci. 2014 Jun 5;9:69. doi: 10.1186/1748-5908-9-69 (PMC4088913; doi:10.1186/1748-5908-9-69)
Supplement: Additional file 2 — Instruction sheet for respondents. [file 1748-5908-9-69-S2.docx]

**Additional file 2. Instruction sheet for respondents**

Data entry hardware: by tablet (e.g. Ipad) or computer/workstation

- Tablet: You are able to record and enter into patient records, everywhere in the hospital
- Computer/workstation: You are able to record and enter into patient records on a number of fixed places (e.g. policlinic, OR, and your room)

Practical support: IT helpdesk or one day training

- IT helpdesk: you can the regular IT helpdesk of the hospital for all IT problems, including the one on the EMR
- Training: The hospital provides for an certified trainer who shows you all the options of the system. After the training, the regular IT provides standard support

Attitude of you manager

- Stimulating: your manager emphasizes that proper use of the EMR is important for the quality of the work by the department
- Biding: your manager emphasizes that the use of the EMR should interfere as little as possible with regular work.

Performance feed back

- Monthly overview: you receive a monthly overview by mail including patient numbers, average age of patients, numbers of diagnoses, numbers of complications and scores of satisfaction of patients
- No overview: you will receive no performance feed back

Flexibility interface

- Flexible: you are able to tailor the information you will see on your screen personally (e.g. patient data, lab, medication, images, diagnose etc).
- Static: the system gives a complete overview of all medic data. U will have to scroll to find information

Decision support

- Present: with audio and visual signs you are warned for risky situations (e.g. extreme medication dose, or medication interactions)

Not present: the system has no alarm function for risky situations.
